# Simultaneous Branch Retinal Artery and Central Retinal Vein Occlusion Improved with No Ocular Therapy: A Case Report

**DOI:** 10.3390/tomography9050139

**Published:** 2023-09-19

**Authors:** Livio Vitiello, Giulio Salerno, Alessia Coppola, Giulia Abbinante, Vincenzo Gagliardi, Alfonso Pellegrino

**Affiliations:** Eye Unit, “Luigi Curto” Hospital, Azienda Sanitaria Locale Salerno, Polla, 84035 Salerno, Italy

**Keywords:** branch retinal artery occlusion, BRAO, central retinal vein occlusion, CRVO, multimodal imaging

## Abstract

A rarely described condition known as branch retinal artery occlusion (BRAO) with concurrent obstruction of the central retinal vein (CRVO) is characterized by diffuse retinal hemorrhages, dilated and tortuous retinal veins, macular and disc edema, cotton wool spots, and a generalized delay in arteriovenous transit on fluorescein angiography, together with a retinal whitening in the area of the affected retinal arterial branch. Although BRAO and CRVO may share underlying systemic risk factors, the pathogenesis of combined BRAO + CRVO is still unknown. We present a BRAO + CRVO case report concerning a 63-year-old white male who came to our observation complaining of sudden vision loss in his right eye. An increased risk for thrombotic event was revealed in this case, and the patient improved only with systemic anticoagulant therapy and in the absence of ocular therapy. We also explain all the clinical findings that are detectable using different diagnostic devices and analyze the scientific literature for other, similar clinical cases.

## 1. Introduction

Conventionally, retinal vein obstructions are divided into central retinal vein occlusion (CRVO), branch retinal vein occlusion (BRVO), and hemi-central retinal vein occlusion, while retinal artery occlusions are classified into central retinal artery occlusion (CRAO), branch retinal artery occlusion (BRAO), and cilioretinal artery occlusion (CLRAO). The concurrent artery and vein obstruction could be any permutation and combination of the clinical aforementioned entities [1].

In particular, BRAO with concomitant CRVO is an uncommon event [1]. This condition is characterized by retinal whitening in the territory of the affected retinal arterial branch and typical CRVO features, such as diffuse retinal hemorrhages, tortuous and dilated retinal veins, disc and macular edema, cotton wool spots, and a generalized delay in arteriovenous transit on fluorescein angiography [2,3,4].

Despite the fact that BRAO and CRVO share several underlying systemic risk factors, such as diabetes, coagulopathies, systemic arterial hypertension, and atherosclerotic cardiovascular disease [2,3,4,5], the pathophysiology of simultaneous BRAO + CRVO remains unknown.

It has been hypothesized that either CRVO followed by the external compression of a nearby branch retinal artery due to the optic disc swelling, or BRAO followed by a low-flow condition in the central retinal vein, could be the initial event in this condition [1,5].

Considering the pathophysiology of retinal vascular occlusive disease and its vision-threatening nature, early diagnosis is essential to try to best manage such a pathological condition and to ensure maximum visual recovery for the patient. For this reason, a multimodal imaging diagnostic approach with color fundus photography, optical coherence tomography (OCT), and fluorescein angiography is advisable for achieving this purpose [1].

There are several papers in the published scientific literature reporting cases of BRAO + CRVO, managed differently and with different outcomes. In fact, some patients received no therapy [5,6,7,8], others received only systemic therapy [5,9,10,11,12,13], while still others also received ocular therapy [10,14,15,16,17,18,19,20].

In this case report, we present a BRAO + CRVO case report concerning a 63-year-old white male who came to our observation complaining of sudden vision loss in his right eye. We evaluated him using multimodal imaging, and he improved only with systemic anticoagulant therapy and in the absence of ocular therapy. Furthermore, we also explain all the clinical findings that were detectable with different diagnostic devices, and we analyze the scientific literature for other, similar clinical cases.

## 2. Case Description

A 63-year old Caucasian male came to our observation complaining of sudden vision loss in his right eye, after some slight visual defects in the previous two days. He referred to being affected by moderate chronic renal insufficiency and grade IV hydronephrosis in his right kidney. Moreover, he was under treatment for arterial hypertension with nebivolol and olmesartan/amlodipine, for hyperlipidemia with ezetimibe, and for benign prostatic hyperplasia with alfuzosin. On admission, his electrocardiogram was normal, and his blood pressure was 125/70 mmHg.

His best-corrected visual acuity (BCVA), evaluated via Snellen chart, was 20/400 in the right eye and 20/20 in the left eye. The intraocular pressure was found to be 18 mmHg in both eyes, and the anterior segment assessment at slit-lamp examination was unremarkable in both eyes. In addition, the pupils were regular, eucyclic, and normoreactive in both eyes, without relative afferent pupillary defect.

A pale region corresponding to the inferotemporal artery branch course was found during the fundoscopic examination of the right eye, together with numerous retinal hemorrhages, macular edema, increased vascular tortuosity, and optic disk swelling. The diagnosis of combined BRAO + CRVO was confirmed via color fundus photography (Visucam 524, version 6.0, Carl Zeiss Meditec, Jena, Germany) and OCT (Cirrus 5000 AngioPlex, version 11.0, Carl Zeiss Meditec, Germany), which also showed subretinal fluid, intraretinal cysts, and a prominent middle-limiting membrane sign in the inner synaptic portion of the outer plexiform layer, corresponding to areas of paracentral acute middle maculopathy (PAMM).

Anticoagulant therapy with subcutaneous injections of heparin 6000 IU twice daily was immediately started after a nephrological and cardiologic consult, and a fluorescein angiography was also scheduled. The results for homocystinemia and screening for vasculitis were all found to be normal. On the other hand, thrombophilic screening revealed methylenetetrahydrofolate reductase gene heterozygosis and clotting factor XIII gene homozygosis, both causing an increased thrombotic risk. The day after the start of anticoagulant therapy, the BCVA improved to 20/70, with a macular edema decrease at OCT.

**Figure 1:**
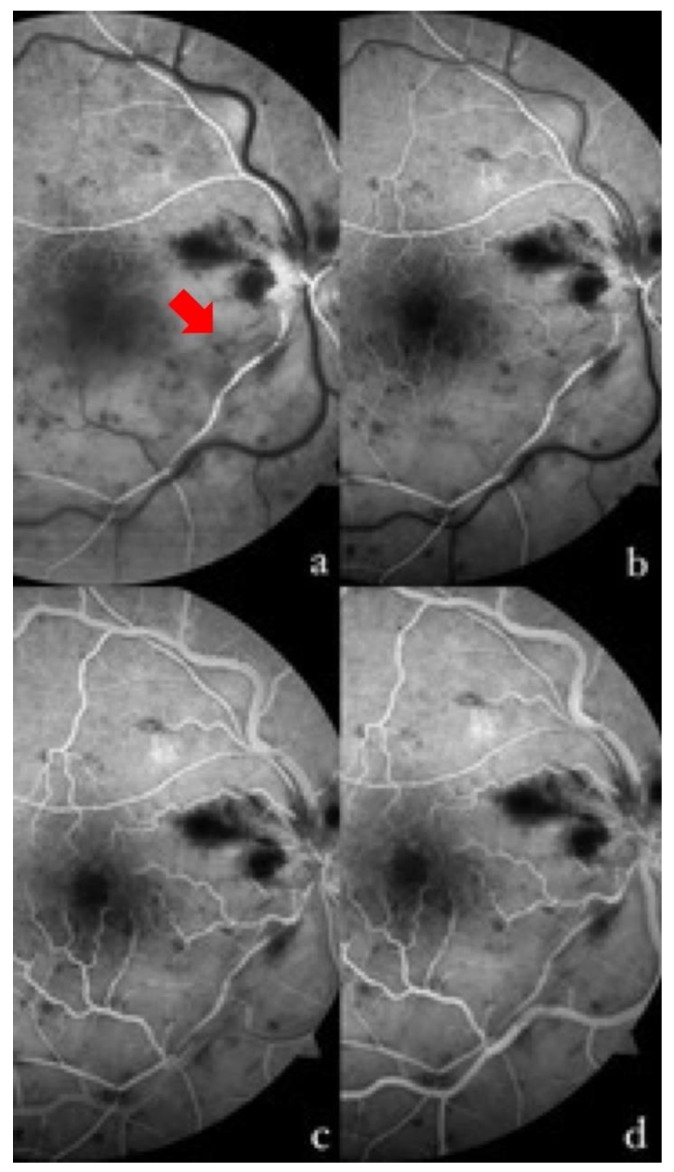
Fluorescein angiography of the right eye at different times in the examination. (**a**) At 13 s, the complete filling of the supero-temporal arterial branch is shown, with the incomplete and delayed filling of the infero-temporal branch and some segments (red arrow). (**b**) At 16 s, the presence of a capillary network at the posterior pole in the superior sector, and its absence in the inferior sector, is well shown. The initial laminar filling of the superior veins, and a slight delay in the temporal inferior one, can be observed. There is a progressive venous filling that completes at 22 s (**c**) in the upper sectors, and at 36 s (**d**) in the lower sectors. Non-perfused arteries and areas can be seen in the affected retina on the temporal inferior side.

Three days after admission, the BCVA further improved to 20/50, and fluorescein angiography was performed (Visucam 524, version 6.0, Carl Zeiss Meditec, Germany) (Figure 1). The exam confirmed the diagnosis, showing a delayed fluorescence of the infero-temporal arterial branch, with incomplete filling at early and late times and sparing of the other arterial branches, delayed venous filling over the entire retinal field, greater in the inferior sectors, and the cattle-trucking sign of infero-temporal peripheral arterioles. Furthermore, the fluorescein angiography revealed an early hypofluorescence for superficial and deep retinal hemorrhages throughout the evaluated retina (mask effect), with areas of capillary nonperfusion (vascular dropout) in the inferior sectors and venous pruning, a fovea avascular zone size increase in the inferior sectors due to the presence of areas of nonperfusion, and the absence of late macular leakage.

Two weeks after the start of anticoagulant therapy, the BCVA further improved to 20/25, with the total resolution of the macular edema and the persistence of a slight hyper-reflectivity at the OCT corresponding to the area of branch retinal arterial occlusion.

**Figure 2:**
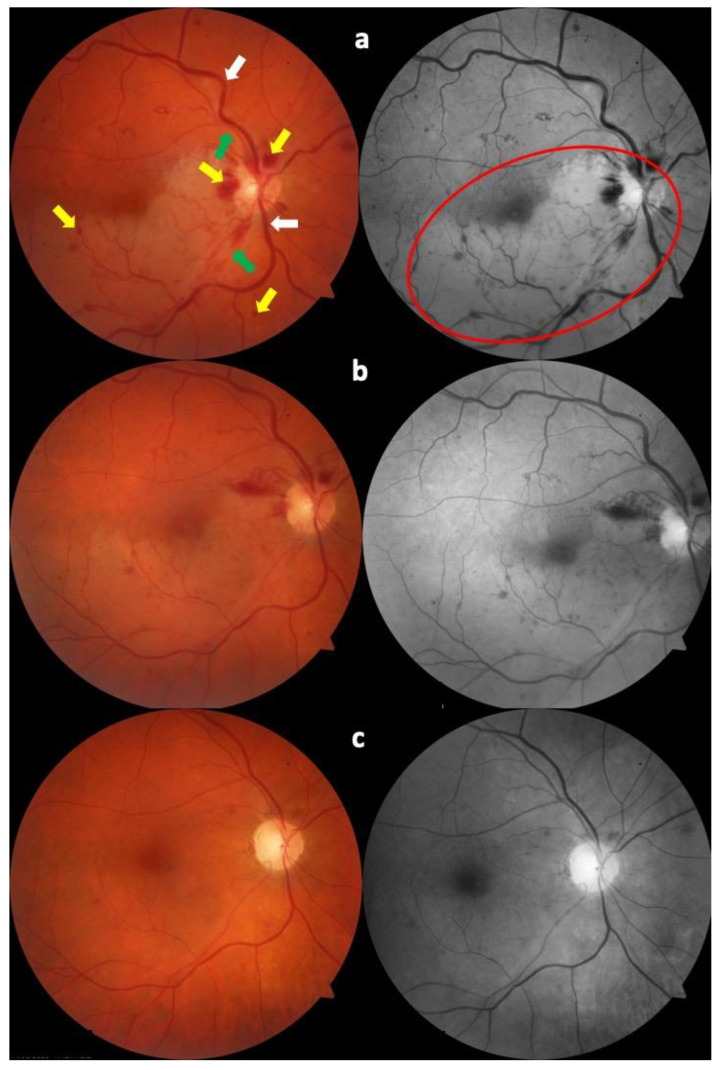
Color fundus photography at diagnosis (**a**). It is possible to see retinal hemorrhages (yellow arrows), temporal retinal veins (white arrows), and temporal retinal arteries (green arrows), with the pale area corresponding to the area of branch retinal artery occlusion (red circle). The same fundus is shown one week later (**b**), and six weeks after diagnosis (**c**). The progressive improvement in the clinical picture is evident, with the gradual disappearance of retinal hemorrhages and the reduction in retinal whitening.

**Figure 3:**
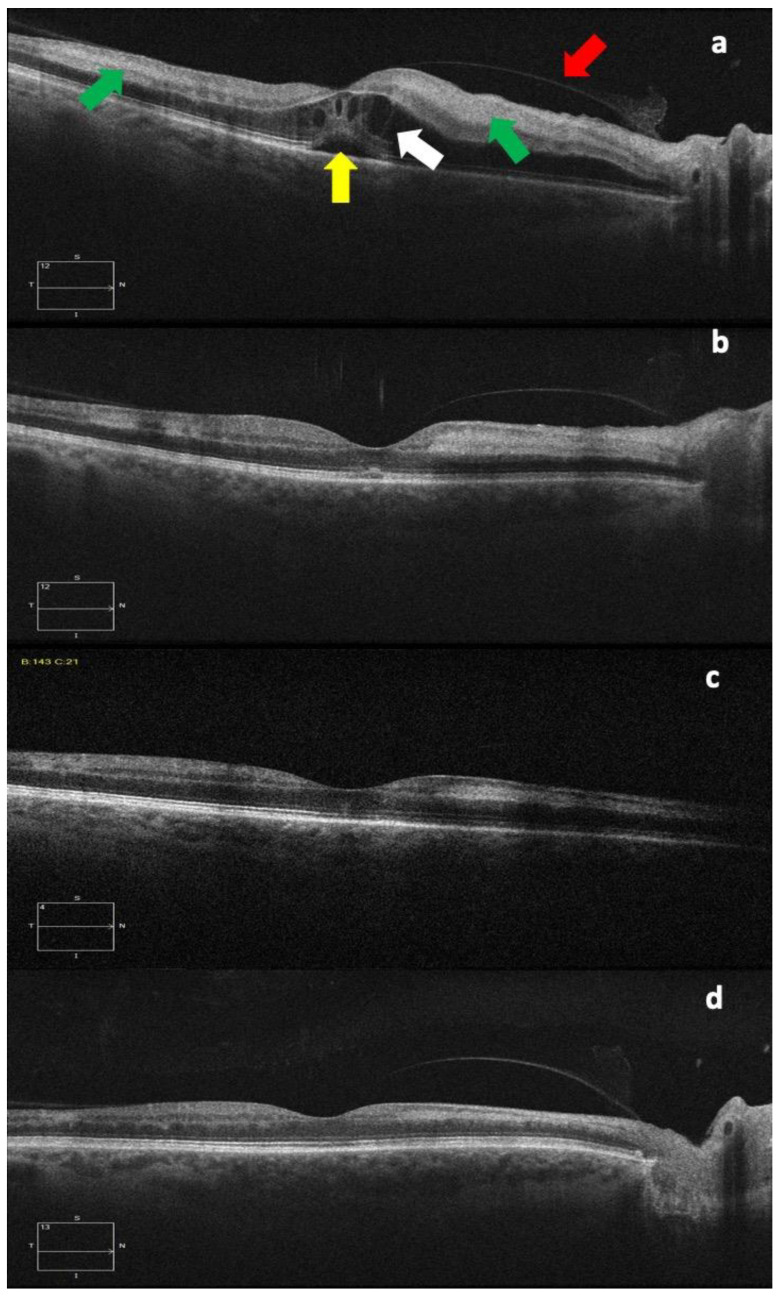
OCT images at diagnosis (**a**), and one week (**b**), three weeks (**c**), and six weeks (**d**) after diagnosis. The different images show the progressive subfoveal (yellow arrow) and intraretinal (white arrow) fluid decreasing with the gradual reduction in the retinal thickness and also the progressive reduction in the hyperreflectivity of the internal retinal layers, corresponding to the area of paracentral acute middle maculopathy (green arrows), an OCT sign that could be typical of retinal arterial occlusions. The red arrow indicates the posterior hyaloid.

Six weeks after diagnosis, the BCVA was 20/20, with a further improvement in the color fundus photography and OCT, as can be seen in Figure 2 and Figure 3.

## 3. Discussion

Combined vascular occlusion affecting the retinal vein and artery is a very infrequent occurrence [1].

In particular, BRAO with concurrent CRVO is a rarely described condition [5,6,7,8,9,10,11,12,13,14,15,16,17,18,19,20]. A number of pathogenic processes have been proposed to explain these simultaneous occlusions. For example, CLRAO might be the result of a rapid rise in intraluminal retinal capillary bed pressure caused by CRVO [2]. Similarly, a reduction in cilioretinal and retinal artery perfusion pressure may result in reduced retinal circulation and consequent venous stasis and thrombosis. Many systemic co-morbidities linked with retinal vein occlusions (e.g., atherosclerosis, systemic arterial hypertension, diabetes mellitus, hyperlipidemia, hyperviscosity, blood diseases, coagulopathies, and systemic vasculitis) can also produce combined vascular occlusion [1].

Concurrent BRAO + CRVO is a clinical condition different to CRVO linked with CLRAO [2,3]. Unless extremely high-quality fluorescein angiography with an early frame is performed in eyes with a cilioretinal artery, it can be difficult to separate the two retinal vascular diseases. In fact, fluorescein angiograms of patients with combined CLRAO + CRVO examined early after onset reveal a distinctive oscillating blood column in the cilioretinal artery. The pathogenesis of concurrent CLRAO + CRVO is believed to be caused by the transient hemodynamic blockage of the cilioretinal artery due to a sudden sharp rise in intraluminal pressure in the retinal capillary bed above the level of the cilioretinal artery, while the exact mechanism of combined BRAO + CRVO is still unknown [2].

Symptomatic, acute, simultaneous BRAO + CRVO, like BRAO, is an urgent ocular disease that requires early systemic assessment. Indeed, BRAO + CRVO may be a critical clinical event in an inflammatory, embolic, or other process necessitating a prompt and focused systemic medical examination based on the patient’s presentation and medical history. Overall, in younger patients (those under the age of 50), a thorough workup for hypercoagulability or vasculitis is required [21], while an embolic workup is recommended for patients above the age of 50 [22].

In this case report, we described a BRAO + CRVO that occurred in a 63-year old Caucasian male and that clinically improved only with systemic anticoagulant therapy, with no need for ocular therapy.

In the scientific literature, there are some similar case reports published on this topic, showing different outcomes and therapeutic approaches, basically due to the lack of official guidelines [5,6,7,8,9,10,11,12,13,14,15,16,17,18,19,20].

Concerning the diagnosis, color fundus photography, OCT, and fluorescein angiography were all demonstrated to be crucial in the identification and the monitoring of this rare ocular vascular syndrome [1]. In particular, in addition to the typical findings detectable via color fundus photography and fluorescein angiography (CRVO features, such as diffuse retinal hemorrhages, tortuous and dilated retinal veins, disc and macular edema, cotton wool spots and a generalized delay in arteriovenous transit on fluorescein angiography, and retinal whitening in the territory of the affected retinal arterial branch) [2,3,4], PAMM is an early OCT characteristic in eyes with concurrent BRAO + CRVO [16,17,18,19,20].

PAMM is an OCT finding that appears as a placoid, hyperreflective band at the level of the inner nuclear layer, with or without extension into the adjacent inner and outer plexiform layers, in individuals with retinal capillary ischemia and unspecific persistent scotomas [23,24]. This OCT feature indicates retinal infarction caused by hypoperfusion within the deep vascular complex and, more specifically, the deep retinal capillary plexus. As this condition resolves, a legacy of inner nuclear layer thinning can develop [23]. It can occur as a standalone event or as a result of an underlying retinal vasculopathy, such as a central artery or vein occlusion [21,25]. As a result, OCT can aid in the identification of the involved regions and in the identification of the underlying condition.

However, regardless of the usefulness of color fundus photography and OCT, fluorescein angiography remains a fundamental diagnostic examination in the diagnosis of retinal vascular and ischemic disease and, for this reason, it should always be performed in cases of BRAO + CRVO suspicion to confirm the diagnosis [1].

In recent years, OCT angiography has gained an increasing usefulness in ischemic retinal diseases diagnosis and management, due to technological updates. In fact, OCT angiography has been demonstrated to correlate visual impairment with the extent of deep capillary plexus involvement in cases of PAMM related to CRVO [26]. Although we did not perform OCT angiography on our patient due to the unavailability of the angiographic software, we believe that this diagnostic technique could further improve the understanding of the BRAO + CRVO pathophysiology in the future.

Regarding management and therapeutical approaches, only one major case series of combined BRAO + CRVO has been published, by Duker et al. in 1990 [5], who described seven individuals with this rare ocular disease. The authors divided the patients into two groups: three patients with no underlying systemic risk factors and four patients with vasculopathic risk factors. In the first group, the visual prognosis was satisfactory (20/30 or better); in two cases, no therapy was utilized, while one patient received intravenous methylprednisolone. On the other hand, two patients in the second group showed significant improvements in visual acuity following treatment with oral acetylsalicylic acid alone or in combination with a carbonic anhydrase inhibitor (20/20 and 20/40, respectively).

In addition, some authors [6,7,8] described the case reports of five patients (four females, one male) with BRAO + CRVO who received no therapy and showed no visual acuity improvement, except for one female patient [8] who showed an improvement in BCVA, from 20/60 to 20/40, six weeks after the onset of symptoms.

On the other hand, the intravitreal dexamethasone implant (Ozurdex) could be considered a useful therapeutical approach, as it has been proven to be effective in the treatment of all subtypes of CRVO [27]. In fact, Ozturk et al. [17] and Arrigo et al. [20] reported that a dexamethasone implant resulted in an excellent visual recovery in two patients with concurrent BRAO + CRVO and macular edema. Similarly, another patient’s visual acuity was reported to have improved after receiving a triamcinolone acetonide periocular injection [16]. Overall, these authors came to the conclusion that the visual prognosis depends on the early treatment of BRAO + CRVO-associated macular edema. Additionally, it has been suggested that intravitreal steroids may lessen macular edema, which would reduce venous engorgement and, as a result, improve arterial perfusion [20].

Nonetheless, the patient in this case report showed a great visual recovery in the absence of any ocular treatment and with only systemic anticoagulant therapy. This result agrees with several case reports already published in the scientific literature [5,9,10,11,12,13].

In addition to the three patients examined by Ducker et al. [5] and treated with oral acetylsalicylic acid, Tavola et al. [9] described a case of BRAO + CRVO in the right eye of a 45-year-old white woman with inherited type I plasminogen deficiency and permanent elevation of lipoprotein(a) that was treated with warfarin for three months and resulted in an improvement in the patient’s BCVA from 20/20,000 to 20/2000.

Rubio et al. [10] reported a 51-year-old man with chronic hepatitis C who was receiving interferon α and ribavirin treatment when his left-eye vision suddenly and painlessly diminished. After he took an oral aspirin, this patient’s BCVA did not improve, and the authors hypothesized that he had an interferon-associated retinopathy, which would have caused significant and irreversible vision loss.

On the other hand, a similar case report was described by Jenisch et al. [12], who examined a 44-year-old female, affected by multiple sclerosis and under treatment with interferon β, who developed a combined BRAO + CRVO, but her BCVA improved from 20/500 to 20/25 after six months of oral aspirin. The authors hypothesized that the presence of an identical ligand involved in signal transduction could determine a similar pathophysiological mechanism and possible similar side effects with the two different forms of interferons [12]. However, the occurrence of unilateral combined BRAO + CRVO without the presence of cardiovascular or thromboembolic risk factors in their patient could suggest a connection with existing interferon β therapy, despite many years of uncomplicated treatment, but idiopathic combined vascular occlusion cannot be ruled out [12].

Özdek et al. [11] evaluated two young men with simultaneous BRAO + CRVO, associated with increased homocysteinemia in their right eye, whose BCVA improved from 20/60 and 20/50 to 20/20 and 20/25 after six months and five months of oral folic acid, respectively. The investigation into elevated homocysteinemia as a risk factor for concurrent BRAO + CRVO in the absence of other risk factors was also emphasized by the authors [11].

Finally, Parchand [13] studied a 30-year-old female with blurred vision in her right eye. Fundoscopy revealed a mixed non-ischemic CRVO and supero-temporal BRAO with a visual acuity of 20/80. Raised blood homocysteine levels were discovered after extensive systemic tests and a heart workup. Pyridoxine and folic acid orally were started, and her visual acuity increased to 20/40 during the following six months as the retinal whitening and hemorrhages in the retina cleared up.

Regarding our case report, the patient showed an increase thrombotic factor due to methylenetetrahydrofolate reductase gene heterozygosis and clotting factor XIII gene homozygosis, in addition to arterial hypertension and hyperlipidemia, which were already being treated with drugs. Moreover, the patient was not taking any type of medication that could further increase the risk of vascular occlusive pathology, as demonstrated for interferon [10,12]. As proposed by Jenisch et al. [12], BRAO + CRVO can certainly be promoted by risk factors, but can also be idiopathic, as seems to have been the case for the patient in this report. For this reason, as already pointed out by Ozdek et al. [11], prevention of, and screening for, any condition favoring vascular occlusive diseases, as well as periodic eye examinations to detect this disease early, become essential.

Concerning systemic anticoagulant therapy, its use in CRVO is controversial. In fact, as explained by Hayreh, the use of antiplatelet aggregation agents or anticoagulants could markedly increase retinal hemorrhages, which can be destructive to the retina [28]. Furthermore, CRVO could also improve spontaneously, as already discussed in this case report. However, our patient had several risk factors for thrombotic events and developed a different clinical entity to CRVO alone, so systemic anticoagulant therapy could be useful, as indicated in this case, even if further studies are needed to better understand its pathophysiological mechanism.

To summarize, this case report confirms that simultaneous BRAO + CRVO is a separate and complex clinical entity that often manifests as abrupt, painless visual loss. In our opinion, according to other authors’ hypothesis [1,5], a possible explanation for this clinical condition could be an involvement of the branch retinal artery secondary to the CRVO. In fact, if vascular crowding is present at the level of the optic nerve head and along its vascular pedicle, an increased venous turgor and associated inflammatory edema may lead to the compression and narrowing of adjacent blood vessels, including the branch retinal artery.

## 4. Conclusions

To establish the right BRAO + CRVO diagnosis, an ophthalmoscopic examination or color fundus photography combined with fluorescein angiography and OCT is required. Although there is currently inadequate information to suggest any particular treatment to enhance vision in concurrent BRAO + CRVO, starting systemic anticoagulant therapy as soon as possible, potentially combined with ocular therapy, appears to comprise a viable treatment approach, especially in case of thrombotic risk factors, as also demonstrated by previous case reports published in the literature [5,6,7,8,9,10,11,12,13,14,15,16,17,18,19,20].

However, it is very important to consider that the CRVO component could also improve spontaneously or after the prompt treatment of extreme hypertension, so every single case should be accurately evaluated.

In conclusion, acute, symptomatic and concurrent BRAO + CRVO, like BRAO, is an urgent ocular disorder that requires early systemic medical assessment to rule out microembolism, hypercoagulability, or vasculitis. Future research is needed to better understand this unusual retinal vascular condition and to draw up useful official treatment guidelines.

## Data Availability

No new data were created or analyzed in this study. Data sharing is not applicable to this article.

## References

[1] Pinna A., Zinellu A., Serra R., Boscia G., Ronchi L., Dore S. (2023). Combined Branch Retinal Artery and Central Retinal Vein Occlusion: A Systematic Review. Vision.

[2] Hayreh S.S., Fraterrigo L., Jonas J. (2008). Central retinal vein occlusion associated with cilioretinal artery occlusion. Retina.

[3] McLeod D., Ring C.P. (1976). Cilioretinal infarction after retinal vein occlusion. Br. J. Ophthalmol..

[4] Richards R.P. (1979). Simultaneous occlusion of the central retinal artery and vein. Trans. Am. Ophthalmol. Soc..

[5] Duker J.S., Cohen M.S., Brown G.C., Sergott R.C., McNamara J.A. (1990). Combined branch retinal artery and central retinal vein obstruction. Retina.

[6] Singh A.J. (2001). Branch retinal artery obstruction with simultaneous central retinal vein occlusion. Eye.

[7] Nicolò M., Artioli S., La Mattina G.C., Ghiglione D., Calabria G. (2005). Branch retinal artery occlusion combined with branch retinal vein occlusion in a patient with hepatitis C treated with interferon and ribavirin. Eur. J. Ophthalmol..

[8] Goel N. (2021). Concurrent branch retinal artery occlusion in central retinal vein occlusion: 3 cases reports and literature review. Saudi J. Ophthalmol..

[9] Tavola A., D’Angelo S.V., Bandello F., Brancato R., Parlavecchia M., Safa O., D’Angelo A. (1995). Central retinal vein and branch artery occlusion associated with inherited plasminogen deficiency and high lipoprotein(a) levels: A case report. Thromb. Res..

[10] Rubio J.E., Charles S. (2003). Interferon-associated combined branch retinal artery and central retinal vein obstruction. Retina.

[11] Özdek Ş., Yülek F., Gürelik G., Aydin B., Hasanreisoğlu B. (2004). Simultaneous central retinal vein and retinal artery branch occlusions in two patients with homocysteinaemia. Eye.

[12] Jenisch T., Dietrich-Ntoukas T., Renner A.B., Helbig H., Gamulescu M.A. (2012). Kombinierter retinaler arteriovenöser Verschluss unter Interferon-β-Therapie [Combined retinal artery and vein occlusions associated with interferon beta therapy]. Ophthalmologe.

[13] Parchand S.M. (2016). Combined central retinal vein and branch retinal artery occlusion in hyperhomocysteinaemia. BMJ Case Rep..

[14] Bajaire B.J., Paipilla D.F., Arrieta C.E., Oudovitchenko E. (2011). Mixed vascular occlusion in a patient with interferon-associated retinopathy. Case Rep. Ophthalmol..

[15] Watanabe M., Ogasawara S., Takahashi A., Takada J., Tanaka Y., Okuwaki Y., Minamino T., Hidaka H., Nakazawa T., Shibuya A. (2012). Branch retinal artery occlusion and central retinal vein occlusion associated with pegylated interferon plus ribavirin combination therapy for chronic hepatitis C. Cutan. Ocul. Toxicol..

[16] Karapetyan A., Ouyang P., Tang L.S., Zeng J., Ying M.D. (2014). Detection of underdiagnosed concurrent branch retinal artery occlusion in a patient with central retinal vein occlusion using spectral domain optical coherence tomography. BMC Ophthalmol..

[17] Ozturk T., Takes O., Saatci A.O. (2015). Dexamethasone implant (Ozurdex) in a case with unilateral simultaneous central retinal vein and branch retinal artery occlusion. Case Rep. Ophthalmol..

[18] Coca M., Tecle N., Made W., Mehta A. (2017). Combined Central Retinal Vein and Branch Retinal Artery Occlusion Post Intense Physical Activity. Cureus.

[19] Raval V., Nayak S., Saldanha M., Jalali S., Pappuru R.R., Narayanan R., Das T. (2020). Combined retinal vascular occlusion: Demography, clinical features, visual outcome, systemic co-morbidities, and literature review. Indian. J. Ophthalmol..

[20] Arrigo A., Knutsson K.A., Rajabjan F., Augustin V.A., Bandello F., Parodi M.B. (2021). Combined central retinal vein occlusion and branch retinal artery occlusion treated with intravitreal dexamethasone implant: A case report. Eur. J. Ophthalmol..

[21] Flaxel C.J., Adelman R.A., Bailey S.T., Fawzi A., Lim J.I., Vemulakonda G.A., Ying G.S. (2020). Retinal and Ophthalmic Artery Occlusions Preferred Practice Pattern^®^. Ophthalmology.

[22] Flaxel C.J., Adelman R.A., Bailey S.T., Fawzi A., Lim J.I., Vemulakonda G.A., Ying G.S. (2020). Retinal Vein Occlusions Preferred Practice Pattern^®^. Ophthalmology.

[23] Iovino C., Au A., Ramtohul P., Bacci T., AlBahlal A., Khan A.M., Al-Abdullah A.A., Wendel R., Chhablani J., Sadda S. (2022). Coincident PAMM and AMN and Insights into a Common Pathophysiology. Am. J. Ophthalmol..

[24] Sebastiani S., Pellegrini M., Giannaccare G., Sarraf D. (2021). Paracentral acute middle maculopathy associated with phosphodiesterase-5 inhibitor therapy. Retin. Cases Brief. Rep..

[25] Rahimy E., Sarraf D., Dollin M.L., Pitcher J.D., Ho A.C. (2014). Paracentral acute middle maculopathy in nonischemic central retinal vein occlusion. Am. J. Ophthalmol..

[26] Casalino G., Williams M., McAvoy C., Bandello F., Chakravarthy U. (2016). Optical coherence tomography angiography in paracentral acute middle maculopathy secondary to central retinal vein occlusion. Eye (London).

[27] Haller J.A., Bandello F., Belfort R., Blumenkranz M.S., Gillies M., Heier J., Loewenstein A., Yoon Y.H., Jiao J., Li X.Y. (2011). Dexamethasone intravitreal implant in patients with macular edema related to branch or central retinal vein occlusion twelve-month study results. Ophthalmology.

[28] Hayreh S.S. (2005). Prevalent misconceptions about acute retinal vascular occlusive disorders. Prog. Retin. Eye Res..

